# The Effect of Vitamin D on Coronary Atherosclerosis: A Propensity Score Matched Case–Control Coronary CTA Study

**DOI:** 10.3390/jcdd8080085

**Published:** 2021-07-25

**Authors:** Gudrun Feuchtner, Simon Suppersberger, Christian Langer, Christoph Beyer, Stefan Rauch, Theresa Thurner, Guy Friedrich, Wolfgang Dichtl, Gerlig Widmann, Fabian Plank, Fabian Barbieri

**Affiliations:** 1 Department of Radiology, Medical University of Innsbruck, 6020 Innsbruck, Austria; gudrun.feuchtner@i-med.ac.at (G.F.); Simon.Suppersberger@student.i-med.ac.at (S.S.); christian.langer@tirol-kliniken.at (C.L.); stefan.rauch@i-med.ac.at (S.R.); gerlig.widmann@tirol-kliniken.at (G.W.); 2 Department of Internal Medicine III, Medical University of Innsbruck, 6020 Innsbruck, Austria; christoph.beyer@i-med.ac.at (C.B.); guy.friedrich@tirol-kliniken.at (G.F.); wolfgang.dichtl@tirol-kliniken.at (W.D.); fabian.plank@i-med.ac.at (F.P.); 3 Gesundheitszentrum Lanserhof, 6072 Lans, Austria; theresa.thurner@gmail.com; 4 Department of Cardiology, Charité University Medicine, Campus Benjamin Franklin, 12203 Berlin, Germany

**Keywords:** atherosclerosis, vitamin D, propensity score matchmaking, coronary computed tomography angiography, cardiovascular disease prevention, imaging

## Abstract

Background: Vitamin D supplementation may be associated with lower cardiovascular (CV) events, but the data are controversial. It remains speculative whether vitamin D supplementation has a direct effect on coronary atherosclerosis. We therefore set out to assess the influence of vitamin D supplementation on the coronary atherosclerosis profile quantified by coronary computed tomography angiography (CTA) in a retrospective case–control cohort study. Methods: 176 patients (age: 62.4 ± 10.4) referred to coronary CTA for clinical indications were included. A total of 88 patients receiving vitamin D supplementation (mean duration 65.3 ± 81 months) were 1:1 propensity score matched with 88 controls for age, gender, smoking, arterial hypertension, positive family history, dyslipidemia, and diabetes. Coronary stenosis severity (CAD-RADS^TM^), mixed plaque burden (weighted for non-calcified), high-risk-plaque (HRP) features, and plaque density (HU) were quantified by CTA. Serum 25-hydroxyvitamin D (OH)-levels were measured in 138 patients and categorized into four groups (0: <20 ng/mL; 1: 20–40 ng/mL; 2: 40–60 ng/mL; and 3: >60 ng/mL) and compared with CTA. Results: The prevalence of atherosclerosis by CTA was similar in both groups (75.6% versus 74.3%, *p* = 0.999), >50% coronary stenosis was slightly higher in controls (*p* = 0.046), but stenosis severity score (CAD-RADS) was not different (*p* = 0.106). Mixed plaque burden (weighted for non-calcified) was lower in patients receiving vitamin D supplementation (*p* = 0.002) and high-risk-plaque prevalence was markedly lower (3.8% versus 32%, *p* < 0.001). CT plaque density (HU) was higher (*p* < 0.001) in the vitamin D group. Patients with serum vitamin D (OH) levels >60 ng/mL had higher plaque density (*p* = 0.04), indicating more calcified and less vulnerable plaque. Conclusions: In this retrospective case–control cohort study, vitamin D supplementation was associated with less high-risk plaque, less non-calcified plaque burden, and a higher calcified plaque independent of CV risk factors.

## 1. Introduction

Vitamin D deficiency has been linked with adverse cardiovascular disease (CVD) outcomes in cross-sectional and prospective studies, while the exact and dominant mechanism has not been fully elucidated [1,2,3,4,5,6,7,8,9,10,11]. A recent meta-analysis of prospective studies including 65,994 patients (24 articles) demonstrated an increase in CVD risk linearly with decreasing 25-hydroxy (OH) vitamin D levels <60 mmol/L [5]. Similarly, in a large cohort of 5559 Koreans, those with lowest serum 25(OH) vitamin D levels <25 nmol/L)] had a two-fold higher rate of CVD as compared to those with higher levels [6]. However, controversy is underlined by studies describing little to no effect of vitamin D supplementation [12,13,14].

Proposed interactions of high vitamin D serum levels with CVD risk include down-regulation of the renin-angiotensin-aldosterone system, a decrease in blood pressure, and improved glycemic control, although supplementation was shown to have no effect on blood pressure control in a recent meta-analysis [1,15]. It is still debatable whether vitamin D mainly modulates via aforementioned indirect mediators or via direct effects on atherosclerosis.

Vitamin D is a prohormone for intestinal calcium absorption; however, its receptor is present in various tissues, including cardiomyocytes, vascular smooth muscle, and the endothelium. Its active form, 1,25-dihydroxy vitamin D (1,25(OH)_2_D_3_), acts as a steroid hormone [2]. Proposed direct mechanisms on coronary atherosclerosis include the improvement of endothelial cell-dependent vasodilatation, the suppression of vascular smooth muscle proliferation, and the stimulation of vascular calcification processes.

In experimental animal models, high dosages of vitamin D supplementation consistently promoted vascular calcification within the medial layer [3]. Effects occurred already within 3–4 days [4] after initiation of therapy. Generally, there are scarce and conflicting data on the correlation of coronary calcification quantified by computed tomography (CT) with 25-hydroxyvitamin D serum levels [7,8]. The first out of two studies found an inverse relationship in a small series of 180 patients [7], but was limited by confounding risk factors, while the second study, enrolling 1131 patients, could not reproduce these results [8]. Beyond this, it is not fully clear whether anti-inflammatory mechanisms are involved [9] in the cardio-protective effects of vitamin D.

To our knowledge, no study to date has investigated the effect of vitamin D supplementation on the coronary artery atherosclerosis profile by quantitative coronary computed tomography angiography (CTA), including stenosis severity [16], total plaque burden, and high-risk “vulnerable” plaque characteristics, which represent novel biomarkers for increased cardiovascular risk [17,18] and myocardial ischemia [19,20]. Total plaque burden by CTA is another predictor of adverse cardiovascular outcomes [21].

Therefore, the aim of our study was to assess the influence of vitamin D supplementation on the coronary atherosclerosis profile by coronary CTA, including high-risk plaque criteria, in a retrospective matched case–control study, and to correlate the serum 25(OH) vitamin D level with the coronary atherosclerosis profile. 

## 2. Materials and Methods

### 2.1. Study Design 

We included patients referred to coronary CTA for clinical indications (suspected coronary artery disease (CAD) and a low-to-intermediate pre-test probability, defined by an exam of a board certified cardiologist, including symptoms, the cardiovascular risk profile, and other tests such as resting ECG, ECG-treadmill stress test, or echocardiography) between December 2015 and December 2017). The study design was retrospective.

Patients completed a lifestyle questionnaire prior to CTA examination, including whether or not they currently or ever received vitamin D supplementation.

Inclusion criteria were:

- Patients with low-to-intermediate Atherosclerotic Cardiovascular Disease (ASCVD) risk [22], non-specific, atypical, or stable chest pain complaints, and/or suspected coronary heart disease based on other prior tests (ECG-stress test, treadmill, or myocardial perfusion test);
- Availability of conventional coronary risk factors according to standardized ESC criteria: arterial hypertension [23] (systolic blood pressure >140 mmHg or diastolic >90 mmHg), dyslipidemia [24] (total cholesterol >200 mg/dL and high-density lipoprotein (HDL) <40 mg/dL; and/or c-low-density lipoprotein (c-LDL) >160 mg/dL), family history (myocardial infarction or sudden cardiac death in an immediate male relative <55 years or female <65 years), smoker (current or quit within the last 6 months), and diabetes. HDL, total cholesterol, c-LDL, and triglyceride levels were determined as well.

Exclusion criteria were previous percutaneous coronary intervention (PCI), coronary artery bypass grafting (CABG), severe aortic stenosis or other moderate to severe grade valvular disease, renal dysfunction (serum glomerular filtration rate <45 mL/min/1.73 m^2^), pregnancy, age of less than 21 years, high-performing endurance athletes (professionals), and HIV infection.

The primary aim of our retrospective case–control study was to assess differences in the coronary atherosclerosis profile by CTA: patients who currently received vitamin D supplementation were propensity score (1:1) matched for age, gender, body-mass-index and the main 5 risk factors (arterial hypertension, smoking, positive family history, dyslipidaemia, and diabetes) with controls who did not report vitamin D supplementation. Additionally, we screened our coronary CTA database from 2006–2009, consisting of 1431 patients, for those who had serum 25(OH) vitamin D levels measured within 3 weeks prior to CTA, for correlation with CTA results.

### 2.2. Computed Tomography Angiography (CTA)

A non-contrast ECG-gated coronary artery calcium score (CACS) with standardized scan parameters (detector collimation 64 × 1.5 mm; 120 kV, image reconstruction 3 mm/1.5 mm increment) was performed and the Agatston Score [25] was calculated. Coronary CTA was performed with a 128-slice dual source CTA (Definition FLASH^TM^, Siemens, Munich Germany), a detector collimation of 2 × 64 × 0.6 mm, a z-flying spot, and a rotation time of 0.28 s. Prospective ECG-triggering was used in regular heart rates <65 bpm (70% of RR-interval), while in heart rates >65 bpm and irregular rates, retrospective ECG-gating was applied. 

An iodine contrast agent (Iopromide, Ultravist 370™, Bayer, Leverkusen, Germany) was injected intravenously (flow rate 4–6 mL/s + 40 cc saline chaser), triggered into arterial phase (bolus tracking; 100 Hounsfield Units (HU) threshold; ascending aorta). The amount of contrast volume 65–120 cc was set according to the individual patient characteristics. Axial images were reconstructed with 0.75 mm slice width (increment 0.4/medium-smooth kernel B26f) during best diastolic and systolic phase.

### 2.3. CTA Image Analysis

**Figure 1:**
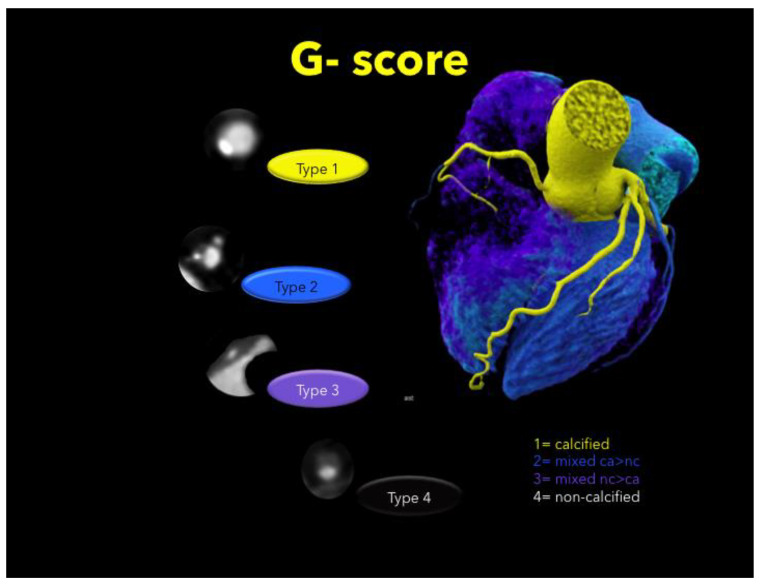
Total plaque burden weighted for non-calcified plaque (G-score). The sum of plaque type 1–4 was calculated for each AHA segment (16-segment classification).

Curved and oblique multiplanar reformations of all vessels by using 3-D postprocessing software (SyngoVia^TM^, Siemens, Munich Germany) were generated:

(1) Coronary stenosis severity was scored on a 5-point scale as: (1) minimal (<25%), (2) mild (25–49%); (3) intermediate (50–69.9%); (4) severe (≥ 70%); and (5) occluded (100%) according to the standardized Coronary Artery Disease Reporting and Data System (CAD-RADS^TM^) classification [16] per-coronary segment (AHA-modified-16-segment classification) [26].
(2) Plaque types were defined as: calcified (T1), mixed (dominantly calcified > non-calcified, T2), mixed (dominantly non-calcified > calcified, T3), or non-calcified (T4) per coronary segment. Calcified and non-calcified plaque were defined as hyper-and hypoattenuating lesions with more and less than 150 HU [27], respectively. Total plaque burden was expressed by the coronary segment involvement (SIS) score [28], and the total mixed plaque burden weighted for the non-calcified plaque component was calculated as previously described [21] (sum of plaque types T1–4 for each segment, G-score) (Figure 1), per-coronary segment (AHA-modified-16-segment classification) [26].
(3) Quantitative High-risk plaque analysis [17,29,30]:
  - Low attenuation plaque, hypodense to the artery lumen, was screened by utilizing the “pixel-lens” for the lowest CT-density (HU) area [18], and then a ROI was drawn as large as possible, while sparing areas affected by motion, beam hardening or partial volume artifacts. The HU ROI was measured on 3 consecutive images (1 mm slice thickness). Low attenuation plaque was defined as “non-calcified” if density was below 150 HU [27];
  - Napkin Ring Sign was defined [17] as low attenuation plaque with a hyperdense rim and hypodense LAP core;
  - Spotty calcification was defined as calcification <3 mm size;
  - The remodeling index was calculated as the ratio of the maximal cross-sectional vessel diameter, including the plaque and the lumen, and its closest proximal (or distal: in ostial lesions) normal reference vessel lumen diameter.

High-risk plaque was identified, if 2 or more of above mentioned 4 criteria were present (according to label ”V“–CAD-RADS^TM^) [16]. For low attenuation plaque, a threshold of 60 HU was set as high-risk plaque criterion [30]. In case of multiple lesions, all were quantified, and the number per patient was recorded.

Computed tomography angiography image analysis was performed by one experienced observer (>10 years’ experience) and a second independent observer with more than 6 months of training. Consensus reading was obtained.

### 2.4. Statistical Analysis

Quantitative variables are expressed as means ± standard deviation, categorical variables as absolute values, and percentages. To reduce possible selection bias and potential confounding, a propensity score matchmaking model was calculated. Therefore, a binary regression was conducted including age, gender, body-mass-index, and the 5 major risk factors (arterial hypertension, smoking, positive family history, dyslipidemia, and diabetes). Given probabilities were matched by using a 1:1 nearest neighbor matchmaking process without replacement, utilizing a matching tolerance of 0.05.

Differences in all parametric data between the 2 groups were tested by using the independent t-test or Mann–Whitney U test, according to their distribution (t-test for normally distributed, Mann–Whitney U test for non-normally distributed, and rank-scale data such as SIS, G-score, CACS, and CAD-RADS^TM^ score). Normal distribution was tested with Kolmogorov’s test and inspection of histograms. Differences in categorical data were determined with Chi-Square or the Fisher’s exact test, if there were less than 5 counts per group. Differences in rank-scale data were tested with the Kruskal–Wallis test. Statistical analysis was performed using IBM SSPS™ software (Version 26, IBM Corporation, Armonk, NY, USA). A *p*-value ≤ 0.05 was considered as significant.

## 3. Results

From our database consisting of 2888 patients who underwent CTA between 2015 and 2017, 88 patients who reported vitamin D supplementation were propensity score (1:1) matched to 88 controls without vitamin D supplementation for age, gender, and the major five cardiovascular risk factors. Twelve patients were excluded, six in the vitamin D group (three due to prior PCI/stent, two due to missing CTA (only CACS performed), and one patient due to HIV positivity) and six in the control group (two due to prior CABG, one due to high-performance professional endurance athleticism, two due to insufficient image quality (high image noise/artifacts), and one due to missing CTA (only CACS performed). Finally, 82 patients for each group were analyzed. Mean vitamin D intake duration was 65.3 ± 81 months (minimum of 2 months up to a maximum of 360 months). 

Table 1 shows the risk profile, age, gender, and laboratory parameters. Generally, there were no significant differences in major risk factors. Only c-LDL levels were slightly higher in the vitamin D group as compared to controls (*p* = 0.020), but the diagnosis of dyslipidemia was not found to be significantly different. Of note, there was a non-significant trend towards a higher rate of arterial hypertension in the control group (*p* = 0.058). 

Table 2 shows the coronary artery disease profile by CTA. The prevalence of coronary atherosclerosis was similar in both groups (*p* = 0.999). Stenosis severity by CTA (CAD-RADS^TM^) was not different between both groups (*p* = 0.106). The proportion of significant (>50%) coronary stenosis by CTA was slightly lower in the vitamin D group as compared to controls (17.1% vs. 31.7%, *p* = 0.046). The CACS was slightly lower in controls without reaching a statistically significant level (*p* = 0.301).

**Figure 2:**
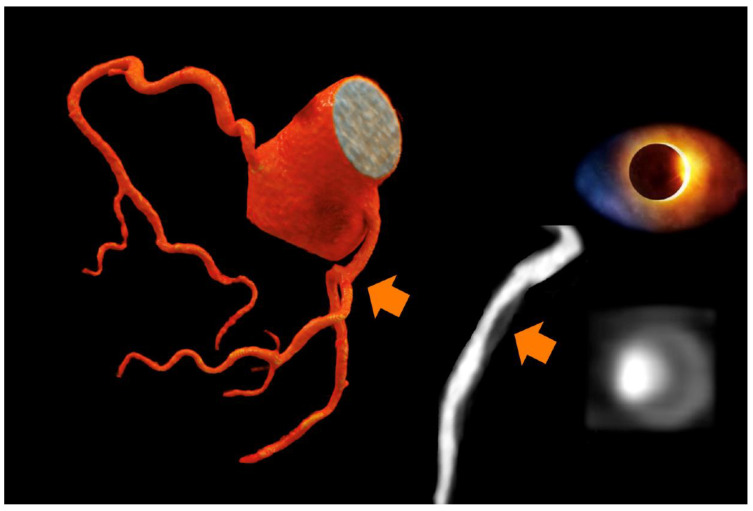
A 67 year-old-male with high-risk plaque (three criteria: low attenuation fibro-fatty plaque with lipid-necrotic core (4 HU), Napkin Ring Sign shown as outer hyperdense rim (lower left panel, orange arrow), and positive remodelling (remodelling index 1.5). Two cardiovascular risk factors (art hypertension and positive family history) and atypical chest pain. No vitamin D supplementation; coronary artery calcium score was zero.

**Figure 3:**
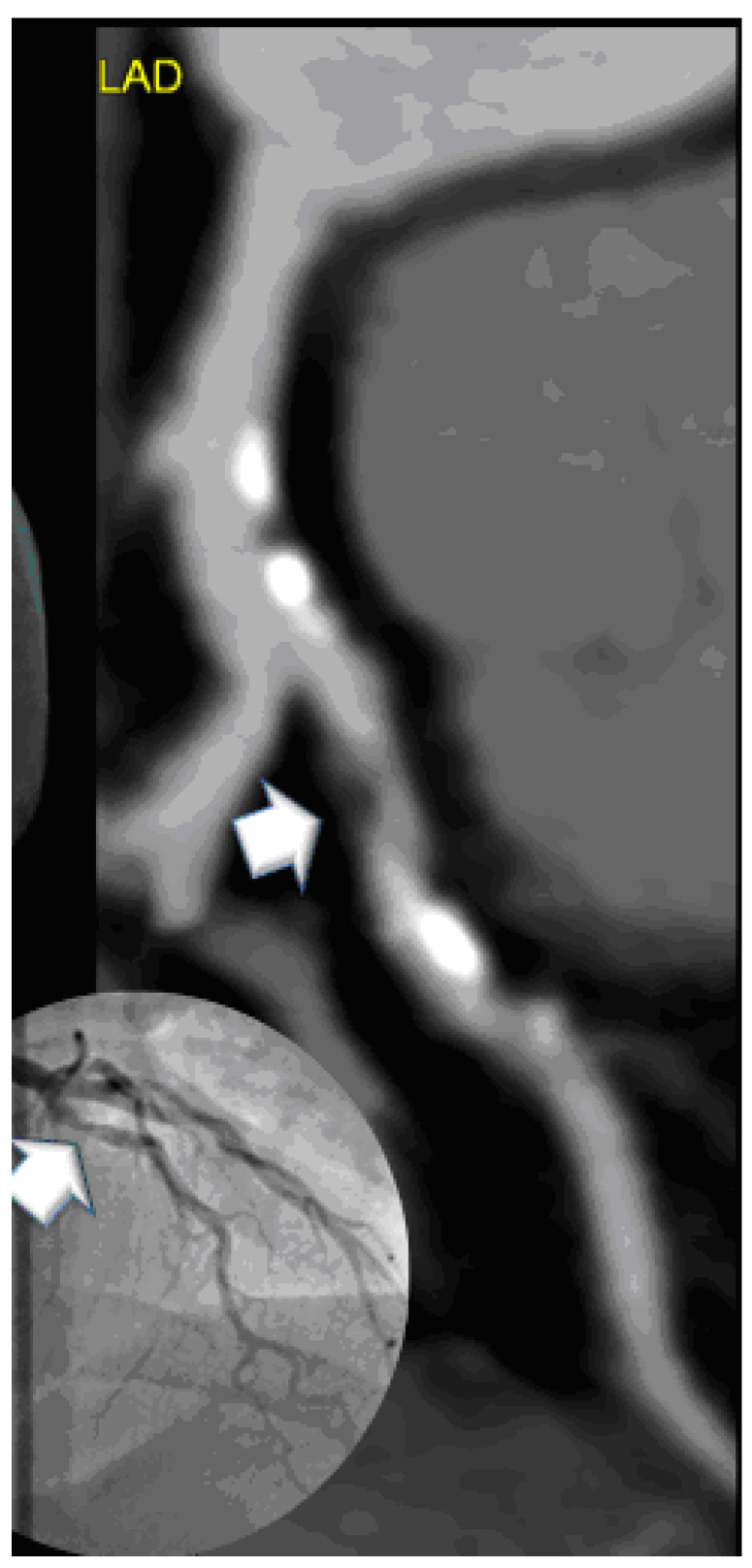
A 49 year-old-male with high grade mid LAD stenosis (>70%), a dense mixed fibrous plaque of 135 HU and more calcifications (white spots). Three cardiovascular risk factors (arterial hypertension, dyslipidemia (total cholesterol 300 mg/dL, high c-LDL of 236 mg/dL), and positive family history) with walking through angina. Vitamin D supplementation for 6 months. Coronary artery calcium score was moderate with 134.2AU. Invasive coronary angiography confirmed stenosis of 80% (inlay bottom left).

High-risk plaques by CTA were less frequently found in the vitamin D group as compared to controls (*p* = 0.012). The high-risk plaque criteria spotty calcification and napkin ring sign were found slightly more often in controls, but prevalence was very low. Figure 2 shows a patient with a high-risk plaque in the control group, while Figure 3 shows a dense mixed plaque and multiple calcified plaques in a patient taking vitamin D as supplementation.

**Figure 4:**
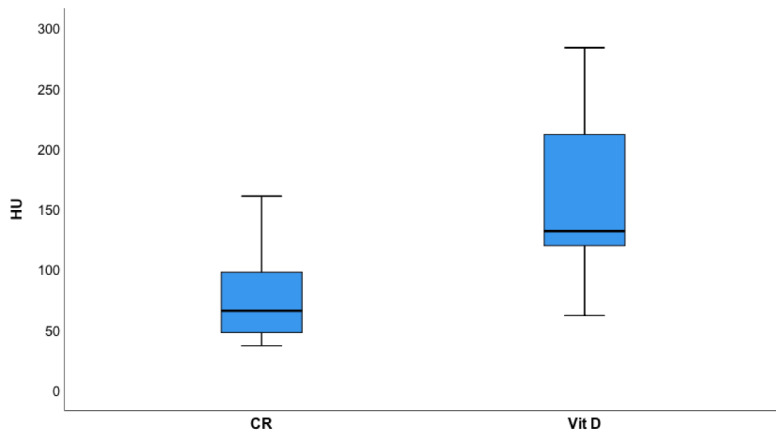
Plaque density (Hounsfield Units, HU) was significantly (*p* < 0.001) higher in those receiving vitamin D (Vit D) supplements as compared to controls (CR) indicating higher calcified plaque component.

**Figure 5:**
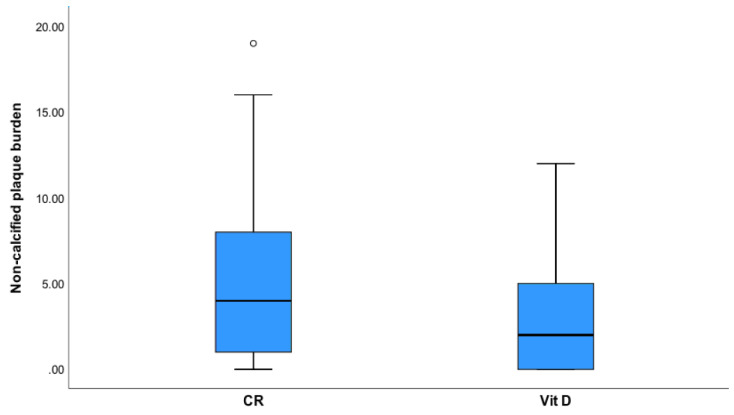
Mixed plaque burden weighted for non-calcified plaque was higher in controls (CR) than in the vitamin D group (Vit D, *p* = 0.002).

The density of plaque (HU) was higher (*p* < 0.001) (Figure 4) in the vitamin D group. Total and mixed non-calcified plaque burden (Figure 5) were lower in the Vitamin D group as compared to controls (*p* = 0.002).

One-hundred thirty six patients had their serum 25(OH) vitamin D levels measured. In total, 25 (OH) vitamin D levels were stratified in four groups: 0 = deficiency (<20 ng/mL): 15 (7.3%); 1 = low (20–40 ng/mL): 28 (20.6%); 2 = moderate (40–60 ng/mL): 28 (20.6%); and 3 = high (>60 ng/mL) 65 (47.8%). There was no difference in stenosis severity (CAD-RADS^TM^) score (*p* = 0.790) among the four groups. When testing thresholds between groups, only patients with a level >60 ng/dL 25 (OH) vitamin D had significantly higher plaque density (HU) (113.11 HU versus 65.44 HU, *p* = 0.041). There was no difference for a threshold of <40 ng/mL (*p* = 0.120) and between the other groups.

## 4. Discussion

These are our three main study findings: (1) Patients who applied vitamin D supplements had markedly less high-risk plaque and (2) lower non-calcified plaque burden. (3) Vitamin D supplementation, and serum 25 (OH) vitamin D levels of >60 mg/dL were associated with higher plaque densities (HU), indicating a higher calcified plaque component.

High-risk plaque are novel imaging biomarkers for the prediction of major adverse events [17,18,28,29,30] and comprise four major criteria: Low attenuation plaque, indicating a lipid-rich necrotic core [18,30]; napkin ring sign (Figure 2); spotty calcification; and positive remodeling. These criteria correlate with plaque vulnerability and inflammation, and represent precursor lesions for acute coronary syndromes [17,18,28,29,30], as shown by other trials [18,31,32].

A prospective randomized multicentre trial [18] identified high-risk plaque as the strongest predictor of MACE, independent of coronary risk factors, coronary artery calcium score, and coronary stenosis severity. As high-risk plaque indicates plaque vulnerability, driven by inflammation, our study findings support direct anti-inflammatory effects of vitamin D on coronary atherosclerosis [9]. This finding was prominent, despite a slightly higher c-LDL level in those under vitamin D supplementation, which is known to increase lipid-rich plaque (LAP <30 HU) [33], most likely explained by prescription bias: Patients with a higher baseline cardiovascular risk are rather advised to intensify preventive measures by as many interventions as possible, including vitamin D supplementation.

Second, less non-calcified plaque burden was observed in those being treated with vitamin D. Higher total plaque burden is a risk factor for MACE and ischemia [19,20], especially when applying a score weighting for the non-calcified plaque burden [21]. Beyond, our study revealed that while stenosis severity score was not different, the proportion of >50% stenosis was slightly higher in those who did not apply vitamin D. However, there was no difference in stenosis severity score (CAD-RADS^TM^) and serum 25-hydroxyvitamin D level.

Studies investigating the direct interaction of vitamin D on coronary atherosclerosis by imaging techniques are scarce. Thus far, only the coronary artery calcification score was applied: In a small Iranian cohort of 180 patients with a mean age of 60 years, an inverse association between coronary calcium and vitamin D level was found. However, this series had major limitations as groups were neither matched for cardiovascular risk factors, nor were all risk factors collected [7]. In a larger and more robust data set of 1131 subjects, Ho et al. reported no association of 25 hydroxyvitamin D levels with coronary artery calcium and the severity of coronary artery disease. However, the number of obstructive diseases (>70% stenosis) by CTA was reported too low to obtain adequate statistical power. [8] We did not find a difference in coronary artery calcium score either, and no difference in the stenosis severity score (CAD-RADS^TM^) was present in our cohort. However, a slightly lower proportion of patients in the Vitamin D group had >50% stenosis by CTA, indicating a trend. The coronary artery calcium score was described in 1990 by Arthur Agatston [25]. Despite the fact that this score detects calcium, it is less accurate as compared to coronary CTA for the detection of calcified plaque due to lower spatial resolution. Coronary CTA has a higher spatial resolution of 0.3–4 mm^2^ [34]. Further, CTA for allows quantification of plaque densities and a more detailed characterization of plaque components. A substantial percentage of patients with early stages of atherosclerosis (up to 25% pending on population characteristics) are not detected by the coronary calcium score [35].

Third, we found higher plaque densities in those under vitamin D supplementation, and in those with a 25-(OH) serum vitamin D level of >60 mg/dL. An increasing plaque density correlates with an increasing calcified plaque component [27]. Rosendaehl et al. reported a decline of acute coronary syndromes along with increasing plaque density in a large multicentre study, with the lowest risk in very dense-compact plaque with >1000 HU (1K-Plaque) [36].

Our study is the first utilizing iodine contrast-enhanced CT-angiography for quantification of plaque characteristics and their relationship to vitamin D. Our results support the cardio-protective effects of vitamin D supplementation, and are in line with a large trial of 10,899 patients, in which vitamin D deficiency was associated with several cardiovascular-related diseases, including hypertension, coronary artery disease, cardiomyopathy, and diabetes. Furthermore, vitamin D deficiency was a strong and independent predictor of all-cause death (OR 2.64, *p* < 0.0001), after adjusting for multiple clinical variables. In addition, vitamin D supplementation conferred substantial survival benefit (*p* < 0.0001) [2].

Overall, the association of vitamin D deficiency with cardiovascular diseases is primarily based on observational and ecological studies, and thus is a matter of controversy. Adequately powered, randomized control clinical trial data would be necessary to confirm these associations. Nevertheless, the available data support adequate vitamin D supplementation for the prevention of cardiovascular disease.

Study limitations comprise the retrospective study design with the possibility of inherent bias (including healthy user bias), and the variable duration of vitamin D supplementation among subjects, with an average dose of 800–1000 IE/day. The c-LDL level was slightly higher in those under vitamin D supplementation, likely due to a prescription bias. Invasive techniques, such as OCT or IVUS, were not performed due to their invasive nature, which would allow an even more detailed investigation of plaque composition. A major implication for performing propensity score matching was controlling confounding factors in a study with rather small population. Nonetheless, its major limitation is the inability to control for unknown confounders, thus potentially leading to biased results.

## 5. Conclusions

Our study supports the cardio-protective effects of vitamin D and confirms a direct effect on atherosclerosis, independent of other cardiovascular risk factors. As high-risk plaque prevalence was lower and plaque density was higher, mainly anti-inflammatory and pro-calcification driving mechanisms seem to be involved, leading to higher plaque density, which may be linked with lower cardiovascular events.

## Figures and Tables

**Table 1:** Baseline characteristics after propensity score matchmaking. Parametric data are shown as means ± SD, ordinal data as counts (*n*) and percentage (%).

	Vitamin D (*n* = 82)	Controls (*n* = 82)	*p*-Value
Age in years	63.2 ± 10.3	61.7 ± 10.5	0.358
Females	57 (69.5%)	51 (62.2%)	0.410
Body mass index in kg/cm^2^	24.9 ± 3.5	26.1 ± 4.6	0.081
Arterial hypertension	29 (35.3)	42 (51.2)	0.058
Smoking	12 (14.6)	18 (21.9)	0.314
Positive family history	47 (57.3)	42 (51.2)	0.531
Dyslipidemia	49 (59.7)	46 (56.1)	0.751
Diabetes	5 (1.2)	7 (8.5)	0.764
Total cholesterol in mg/dL	202.6 ± 55	218.5 ± 45	0.180
Low density lipoprotein in mg/dL	141.1 ± 41	117.2 ± 47	0.020
High density lipoprotein in mg/dL	61.0 ± 18	58.4 ± 19	0.552
Atherosclerotic cardiovascular disease risk	11.8 ± 1.3	11.2 ± 1.4	0.935

**Table 2:** Coronary artery disease profile assessed by computed tomography angiography. Parametric data are shown as means ± SD, ordinal data as counts (n) and percentage (%). ** mean of 3 slices.

	Vitamin D (n = 82)	Controls (*n* = 82)	*p*-Value
Atherosclerosis	62 (75.6%)	61 (74.3%)	0.999
CAD-RADS^TM^			
0	20 (24.4%)	21 (25.6%)	
1	18 (22.0%)	10 (12.2%)	
2	30 (36.6%)	25 (30.5%)	0.106
3	7 (8.5%)	4 (4.9%)	
4/5	7 (8.5%)	22 (26.8%)	
Total >50%	14 (17.1%)	26 (31.7%)	0.046
CAD RADS^TM^	1.54 ± 1.2	1.95 ± 1.5	0.106
Total plaque burden(SIS)	2.7 ± 2.9	3.5 ± 3.0	0.002
Mixed plaque burden (G-score)	2.8 ± 3.2	5.3 ± 5.0	0.002
Coronary artery calcium score (Agatston Units)	69.7 ± 18.1	118.6 ± 264	0.301
High risk plaque	2 (2.4%)	12 (14.6%)	0.012
CT density ROI in Hounsfield Units	158.0 ± 68	75.1 ± 35	<0.001
CT density lens in Hounsfield Units	144.5 ± 68	59.7 ± 36	<0.001
CT density ROI (mean **) in Hounsfield Units	157.9 ± 59	67.2 ± 24	<0.001
Spotty calcification	2 (2.4%)	5 (6.1%)	0.443
Napkin ring sign	1 (1.2%)	4 (4.9%)	0.367

## Data Availability

The data have not been publicly available.
